# TTF2 promotes replisome eviction from stalled forks in mitosis

**DOI:** 10.1101/2024.11.30.626186

**Published:** 2024-11-30

**Authors:** Geylani Can, Maksym Shyian, Archana Krishnamoorthy, Yang Lim, R. Alex Wu, Manal S. Zaher, Markus Raschle, Johannes C. Walter, David S. Pellman

**Affiliations:** 1 Department of Cell Biology, Blavatnik Institute, Harvard Medical School, Boston, MA 02115, USA; 2 Department of Pediatric Oncology, Dana-Farber Cancer Institute, Boston, MA 02215, USA; 3 Department of Biological Chemistry and Molecular Pharmacology, Blavatnik Institute, Harvard Medical School, Boston, MA 02115, USA; 4 Present Address: Arrakis Pharmaceuticals, Waltham, MA, USA; 5 Technische Universitat Kaiserslautern, Kaiserslautern, Germany; 6 Howard Hughes Medical Institute

## Abstract

When cells enter mitosis with under-replicated DNA, sister chromosome segregation is compromised, which can lead to massive genome instability. The replisome-associated E3 ubiquitin ligase TRAIP mitigates this threat by ubiquitylating the CMG helicase in mitosis, leading to disassembly of stalled replisomes, fork cleavage, and restoration of chromosome structure by alternative end-joining. Here, we show that replisome disassembly requires TRAIP phosphorylation by the mitotic Cyclin B-CDK1 kinase, as well as TTF2, a SWI/SNF ATPase previously implicated in the eviction of RNA polymerase from mitotic chromosomes. We find that TTF2 tethers TRAIP to replisomes using an N-terminal Zinc finger that binds to phosphorylated TRAIP and an adjacent TTF2 peptide that contacts the CMG-associated leading strand DNA polymerase ε. This TRAIP-TTF2-pol ε bridge, which forms independently of the TTF2 ATPase domain, is essential to promote CMG unloading and stalled fork breakage. Conversely, RNAPII eviction from mitotic chromosomes requires the ATPase activity of TTF2. We conclude that in mitosis, replisomes undergo a CDK- and TTF2-dependent structural reorganization that underlies the cellular response to incompletely replicated DNA.

## Main Text:

Replisomes encounter many obstacles that have the potential to severely disrupt DNA replication and genome integrity (1). These include transcription complexes, as well as covalent DNA-protein cross-links (DPCs) and DNA inter-strand cross-links (ICLs) that are generated by agents such as endogenous aldehydes and chemotherapeutics. Cells employ various strategies to overcome these challenges. If these strategies fail, cells enter mitosis with under-replicated DNA, triggering additional defense mechanisms to prevent chromosomal instability. These include unwinding (dissolution) or controlled breakage (resolution) of the unreplicated segment (2). The latter pathway is referred to as common fragile site (CFS) expression and involves nucleolytic cleavage of the stalled forks and joining of the resulting broken chromosomes by alternative end-joining (3). Although CFS expression results in a deletion and sister chromatid exchange, it avoids the potentially catastrophic consequences of chromosome non-disjunction.

The E3 ubiquitin ligase TRAIP has been linked to DNA repair in interphase and to the response to under-replicated DNA in mitosis (4). TRAIP is essential for embryonic development and cell proliferation (5)(6)(7), and hypomorphic mutations in TRAIP are associated with dwarfism in humans (8). Growing evidence suggests that TRAIP plays a central role in overcoming replication obstacles in S phase (4). Cells lacking TRAIP are sensitive to agents that induce DNA inter-strand crosslinks (ICLs) and DNA-protein crosslinks (DPCs), and to conditions that promote replication-transcription collisions (9). Indirect evidence suggests that TRAIP binds replisomes with its catalytic RING domain directed ahead of the CMG, allowing it to ubiquitylate “*in trans*” any protein barrier encountered by the replisome (e.g. DPCs), while being unable to ubiquitylate “*in cis*” the replisome with which it travels (fig. S1A; “hood ornament” model)(10, 11). This block to *cis* ubiquitylation is crucial to avoid unscheduled CMG unloading, which would disrupt S-phase DNA replication and lead to permanent fork arrest and genome instability (12)(13)

TRAIP’s interaction with the replisome appears to be much less constrained in mitosis (fig. S1B). In support of this idea, when replisomes stall on either side of a lac repressor (LacR) array in mitotic egg extracts, TRAIP can now ubiquitylate the stalled CMGs with which it travels, leading to p97-dependent CMG unloading, fork breakage, and DNA polymerase θ (pol θ)-mediated alternative end-joining (TMEJ) (14, 15). Furthermore, when CMG unloading in S phase is prevented in cells by neutralizing CRL2^Lrr1^, CMGs are unloaded in mitosis by TRAIP (14)(16). Finally, when the completion of DNA replication is experimentally impaired, TRAIP promotes mitotic DNA synthesis, and it suppresses the formation of anaphase bridges and micronuclei (17). Together with other results (18), these observations suggest that when forks fail to fully converge in S or G2, TRAIP-mediated symmetrical fork cleavage in mitosis restores one chromosome and regenerates the other via alternative end joining, with accompanying sister chromatid exchange and production of a deletion (fig. S1C) (14). This model accounts for key aspects of common fragile site expression and the sequence features associated with common fragile site rearrangements in cancer (3). The TRAIP-dependent unloading of replisomes in mitosis appears to be a last-ditch cellular response to avert anaphase with unreplicated DNA. How TRAIP changes specificity between interphase and mitosis is unclear.

The SWI/SNF ATPase TTF2 was previously shown to promote the eviction of RNAPII from mitotic chromosomes (19). Here, we report an unexpected role for TTF2 in activating the mitotic function of TRAIP. Using Xenopus egg extracts, we show that an N-terminal Zinc finger domain of TTF2 interacts with threonine 325 in TRAIP that has been phosphorylated by B-CDK1. In addition, a short TTF2 peptide that resides adjacent to the TTF2 zinc finger binds to the POLE2 subunit of DNA polymerase ε. These two TTF2 contacts tether TRAIP to the replisome to allow mitotic CMG ubiquitylation and unloading, fork breakage, and alternative end-joining. Surprisingly, the C-terminal ATPase domain of TTF2 is not required to promote TRAIP function. Conversely, in mammalian cells, the TTF2 ATPase domain is sufficient to promote RNAPII eviction in mitosis. Our results indicate that TTF2 uses distinct mechanisms to remove replication and transcription complexes from mitotic chromosomes, and that TTF2 is an essential factor in the TRAIP-dependent pathway that mitigates the threat of incomplete DNA replication.

## Results

### Identification of CDK sites that promote mitotic TRAIP function

**Fig. 1:**
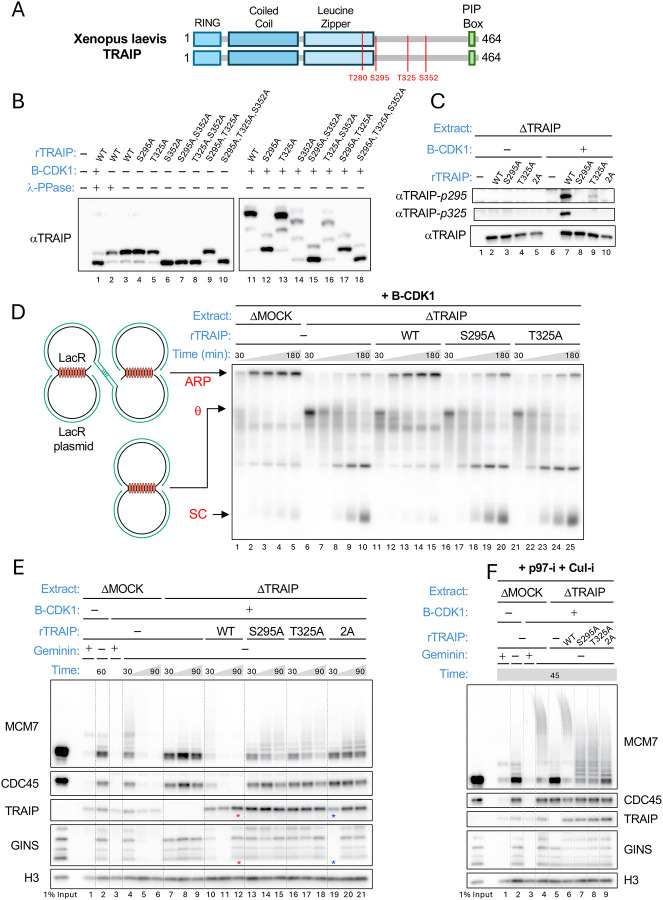
TRAIP is activated by B-CDK1 phosphorylation in mitotic egg extracts. **(A)** Domain organization and conserved CDK sites (red) of Xenopus laevis TRAIP, which is a dimer (21). **(B)** TRAIP is phosphorylated. The indicated TRAIP proteins were translated in wheat germ extracts and added to TRAIP-depleted Xenopus nucleoplasmic extract optionally supplemented with B-CDK1. Samples were treated with λ phosphatase, as indicated, separated on a Phos-tag gel, and blotted for TRAIP protein. **(C)** S295 and T325 are phosphorylated. The indicated TRAIP proteins were added to TRAIP-depleted NPE that was optionally supplemented with B-CDK1. Samples were blotted with the indicated antibodies, including two phospho-specific TRAIP antibodies, whose specificity was validated by the absence of signal in the appropriate phospho-site mutations (lanes 8 and 9). 2A, TRAIP S295A/T325A double mutant. **(D)** TRAIP’s CDK sites are required for ARP formation. A plasmid with 48 tandem *lacO* sites bound to LacR (“LacR plasmid”) was replicated in the indicated egg extracts supplemented with [α−^32^P]dATP and different TRAIP mutants expressed in TTE. Samples were withdrawn at 30, 45, 60, 90, and 180 minutes after replication was initiated and subjected to gel electrophoresis and autoradiography. The structures and positions of ARP, θ, and supercoiled plasmid (SC, enriched when fork cleavage fails) are indicated. **(E)** TRAIP’s CDK sites are required for CMG unloading from stalled forks. LacR plasmid was replicated in the indicated egg extracts supplemented with different TRAIP mutants expressed in TTE, geminin (to prevent licensing and replication initiation), and B-CDK1, as noted. At 30, 45, and 90 minutes, chromatin was recovered and analyzed by blotting with the indicated antibodies. Two chromatin samples that were run on the same gel and blotted for TRAIP and GINS were inadvertently switched (red and blue asterisks). Here and in other complicated gels, samples with related conditions are grouped by dotted lines. **(F)** TRAIP’s CDK sites are required for ubiquitylation of stalled CMGs. LacR plasmid was replicated and recovered as in (E), except extracts contained NMS873 (p97-i) and MLN4924 (Cul-i; to inhibit CRL2^Lrr1^-dependent ubiquitylation), and chromatin was recovered at 45 minutes.

We hypothesized that mitotic TRAIP activation involves its phosphorylation by Cyclin B-CDK1 (B-CDK1). To test this idea, we used frog egg extracts because they faithfully recapitulate TRAIP’s interphase role in repairing DNA lesions and its mitotic role in replisome disassembly (10, 14, 15, 20). TRAIP forms a dimer via its coiled-coil and leucine zipper domains (21)(predictomes.org) and contains four potential B-CDK1 sites (S/TP; Fig. 1A; red residues). Among these, S295, T325 (T324 in human), and S352 (S348 in human) are conserved in vertebrates (fig. S2A). To address whether TRAIP is phosphorylated in egg extracts, we added TRAIP that was expressed in a transcription-translation extract (TTE) to TRAIP-depleted frog egg extracts that were also supplemented with B-CDK1, and separated the proteins on a Phos-tag gel (22). As shown in Fig. 1B, TRAIP underwent a large mobility shift in the presence of B-CDK1 (compare lanes 3 and 11). Mutating serine 352 to alanine increased the mobility of all forms of TRAIP in interphase and mitotic egg extract (Fig. 1B, lanes 6 and 14), indicating that this site is constitutively phosphorylated throughout the cell division cycle. Consistent with this interpretation, S352 corresponds to an ideal CDK phosphorylation site (SPTK) that could be phosphorylated by CDK2 in both extracts. In contrast, mutating S295 dramatically reduced TRAIP mobility in mitosis but not in interphase (Fig. 1B, lanes 4 and 12), and mutating T325 caused a small mobility shift in mitotic but not interphase extract (Fig. 1B, lanes 3 and 5 vs. 11 and 13). To verify that S295 and T325 were phosphorylated, we generated phospho-specific antibodies to both residues (Fig. 1C, lanes 7–9) and showed that both residues were phosphorylated only in extracts supplemented with B-CDK1 (Fig. 1C, lanes 2 vs. 7). Interestingly, mutating S295 abolished phosphorylation at both sites (Fig. 1C, lane 8), whereas mutating T325 greatly reduced but did not eliminate S295 phosphorylation (Fig. 1C, lane 9). These results suggest that B-CDK1 phosphorylates both sites, and that S295 phosphorylation primes T325 phosphorylation whereas S295 can be phosphorylated in the absence of T325 phosphorylation, albeit inefficiently.

We next addressed the potential function of these phosphorylation events. We showed previously that when a plasmid containing a lac repressor array (LacR plasmid) is replicated in frog egg extract containing B-CDK1, replisomes stall at the outer edges of the array, generating a “θ” structure (Fig. 1D, lane 1)(14). TRAIP then promotes CMG ubiquitylation, which leads to replisome disassembly by the p97 ATPase, followed by fork breakage. The broken forks undergo pol θ-mediated end-joining to generate complex, aberrant replication products (ARPs) that are retained in the well of the gel (Fig. 1D, lanes 2–5). As expected (14), immunodepletion of TRAIP from the extract inhibited ARP formation and enhanced appearance of supercoiled products, and re-addition of TRAIP^WT^ rescued these defects (Fig. 1D, lanes 6–15; fig. S2B). In contrast, neither TRAIP^S295A^ nor TRAIP^T325A^ restored ARP formation, indicating that TRAIP phosphorylation at these sites is essential for its mitotic function (Fig. 1D, lanes 16–25; fig. S2B). Consistent with defective ARP formation, the unloading of stalled CMGs that is normally promoted by TRAIP^WT^ in mitotic extracts (Fig. 1E, lanes 10–12)(14) was not supported by TRAIP^S295A^ or TRAIP^T325A^ (Fig. 1E, lanes 13–18; fig. S2C). Accordingly, MCM7 ubiquitylation (measured in the presence of an inhibitor of p97 to prevent CMG unloading) was also greatly diminished in the presence of the phospho-site mutations (Fig. 1F, lanes 6–8; fig. S2C), probably below the threshold normally required for CMG unloading (23)(10). Mutating both S295 and T325 did not further reduce CMG ubiquitylation compared to the single mutants (Fig. 1F, lanes 7–9; fig. S2C), indicating that the two phosphorylation events control the same pathway. In contrast, TRAIP depletion reduced CMG ubiquitylation still further (Fig. 1F, lane 5), suggesting that a basal level of TRAIP-dependent CMG ubiquitylation still occurred in the absence of these phosphorylation events, perhaps due to some residual TRAIP binding to the replisome. However, this residual activity was not sufficient to support efficient CMG unloading (Fig. 1E, lanes 13–21). Importantly, like TRAIP^WT^ (10), both TRAIP^S295A^ and TRAIP^T325A^ supported the unloading of CMGs that have converged on an ICL in interphase egg extracts (fig. S3), indicating that these mutations only abolish the mitotic function of TRAIP. Our results indicate that phosphorylation of TRAIP on at least two sites by B-CDK1 is specifically required for TRAIP function in mitotic egg extracts.

### TTF2 is essential for mitotic TRAIP function

**Fig. 2:**
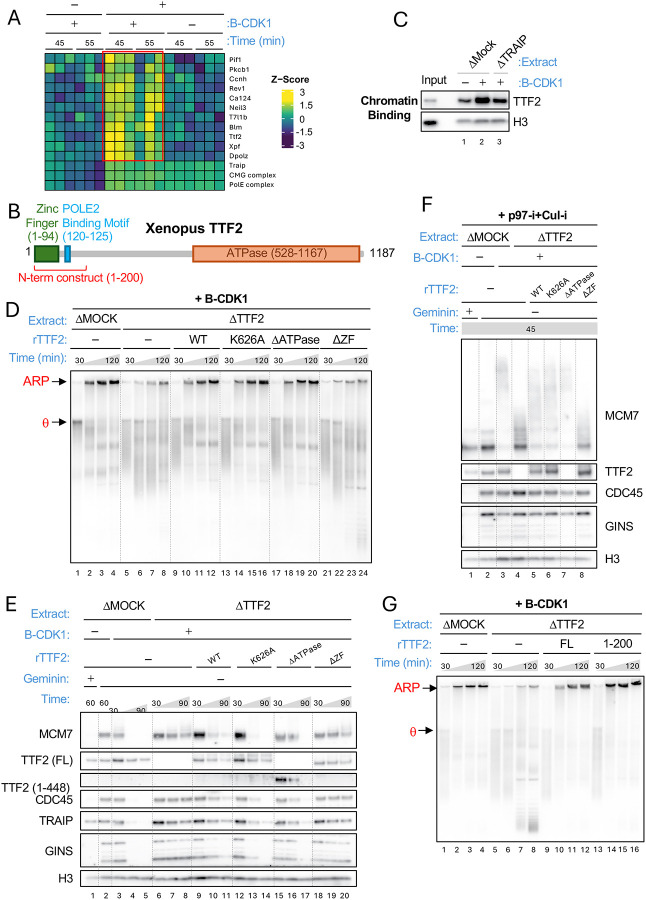
TTF2’s zinc finger domain is required for mitotic CMG ubiquitylation by TRAIP. **(A)** Mass spectrometry analysis of chromatin-associated proteins. LacR plasmid was replicated in the indicated egg extracts (as in Fig. 1D), recovered, and analyzed by label free mass spectrometry. Three replisome components are shown (bottom three rows), as are proteins specifically enriched in the presence of both B-CDK1 and p97-i (red box). For full results and confidence levels, see fig. S4 and Table S1. **(B)** Domain organization and key elements of Xenopus TTF2. **(C)** TRAIP is required for mitotic TTF2 recruitment. LacR plasmid replicated in the indicated extracts (as in Fig. 1D) was recovered (as in Fig. 1E) after 30 minutes, and blotted for TTF2 and histone H3. **(D)** The TTF2 Zinc finger domain but not its ATPase function is required for ARP formation. ARP assay was performed as in Fig. 1D using the indicated extracts and TTF2 constructs. **(E)** The TTF2 Zinc finger domain but not its ATPase function is required for stalled CMG unloading in mitosis. CMG unloading was measured (as in Fig. 1E) using the indicated extracts and TTF2 constructs. **(F)** The TTF2 Zinc finger domain but not its ATPase function is required for CMG ubiquitylation. CMG ubiquitylation was performed (as in Fig. 1F) using the indicated extracts and TTF2 constructs. **(G)** TTF2(1–200) is sufficient to support ARP formation. ARP assay was performed as in Fig. 1D using the indicated extracts and TTF2 constructs.

To identify new proteins that cooperate with TRAIP in mitosis and to explain how TRAIP is activated by B-CDK1, we screened for proteins that specifically associate with mitotic stalled replisomes. To this end, we replicated LacR plasmids in egg extract containing B-CDK1 and p97 inhibitor to accumulate mitotic replisomes, isolated chromatin, and performed mass spectrometry. As controls, we recovered chromatin from reactions lacking p97-i (mitosis with replisome removal) or B-CDK1 (interphase without replisome removal). This analysis revealed a handful of proteins that were only enriched on mitotic chromatin containing replisomes (Fig. 2A, red box and fig. S4). TTF2, which contains a C-terminal SWI/SNF ATPase domain (Fig. 2B), was particularly interesting because it removes RNA polymerase II from mitotic chromosomes (19), a process that is reminiscent of mitotic CMG eviction. Indeed, TTF2 is cytoplasmic during interphase and gains access to chromosomes after mitotic nuclear envelope breakdown (19)(24).

We raised a peptide antibody against Xenopus TTF2 (fig. S5A) and found that the protein’s recruitment to chromatin was enhanced in mitotic extract, in a manner that partially depended on TRAIP (Fig. 2C). Moreover, like TRAIP depletion, depletion of TTF2 from egg extract greatly reduced ARP formation (Fig. 2D, lanes 1–8; fig. S5B), CMG unloading (Fig. 2E, lanes 3–8; fig. S5B), and CMG ubiquitylation (Fig. 2F, lanes 3–4; fig. S5B), and these defects were rescued with recombinant wild type TTF2 (Fig. 2D–F; fig. S5B). Therefore, TTF2 is essential for TRAIP-dependent CMG unloading in mitosis.

We next addressed which domains of TTF2 promote CMG unloading. TTF2 contains an N-terminal Zinc finger domain (ZF; residues 1–94), an internal segment predicted to be largely disordered (residues 95–527), and a C-terminal ATPase domain (residues 528–1167) (Fig. 2B). A point mutation predicted to inactivate the ATPase activity of TTF2 (K626A) supported normal ARP formation, CMG unloading, and CMG ubiquitylation, as did deletion of the entire ATPase domain (Fig. 2D–F; fig. S5B). In contrast, deletion of the Zn finger domain inhibited these activities (Fig. 2D–F; fig. S5B). We also noticed a conserved stretch of amino acids (120–125) located adjacent to the Zn finger (Fig. 2F; blue box). We therefore generated TTF2^1−200^ (Fig. 2B; red bracket), which includes the Zn finger domain and adjacent motif, and found that this construct was sufficient to restore ARP formation (Fig. 2G; fig. S5C). Therefore, the TTF2 Zn finger and adjacent motif, whose importance is documented below, are sufficient for mitotic TRAIP function, whereas, unexpectedly, the ATPase domain is dispensable.

### Evidence that TTF2 interacts with phosphorylated T325 in TRAIP

**Fig. 3:**
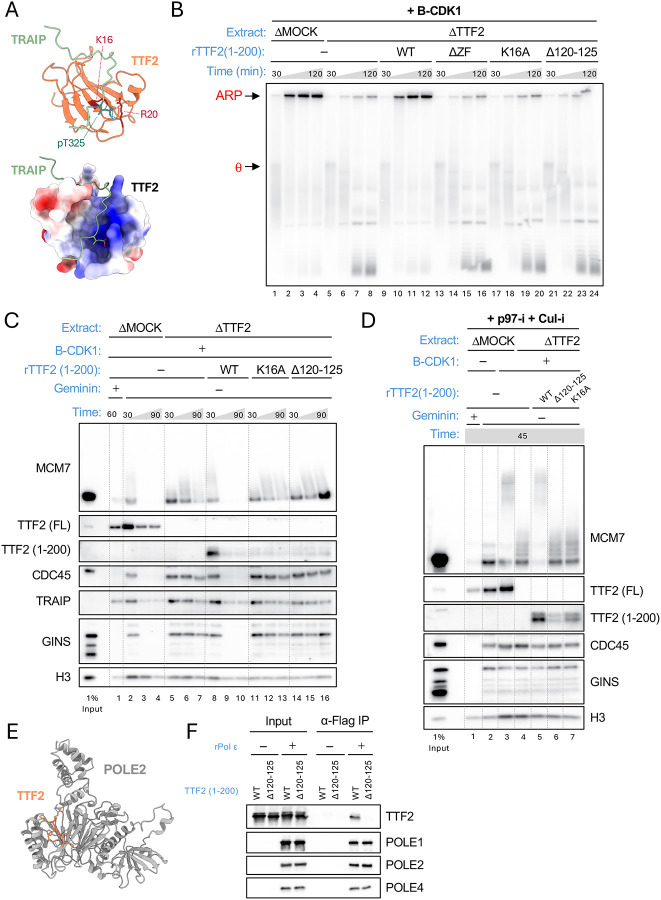
TRAIP and POLE2 binding by TTF2 is required for mitotic CMG unloading. **(A)** AlphaFold3-predicted complex between TRAIP (280–339) containing phosphorylated T325 and TTF2 (1–200). Top, ribbon diagram. Bottom, same orientation as top with TTF2 shown with surface charge representation. **(B)** The predicted binding of TTF2 to TRAIP and POLE2 is required for ARP formation. ARP assay was performed as in Fig. 1D using the indicated extracts and TTF2 constructs. Although deletion of the Zinc finger from TTF2(1–200) abolished ARP formation, this result is not interpretable because we could not assess this mutant’s expression (see fig. S5D). **(C)** The predicted binding of TTF2 to TRAIP and POLE2 is required for CMG unloading. CMG unloading was performed (as in Fig. 1E) using the indicated extracts and TTF2 constructs. **(D)** The predicted binding of TTF2 to TRAIP and POLE2 is required for CMG ubiquitylation. CMG ubiquitylation was performed (as in Fig. 1E) using the indicated extracts and TTF2 constructs. **(E)** TTF2 is predicted to interact with POLE2. AF-M-predicted complex of full length TTF2 and POLE2 (see predictomes.org). Only the interacting peptide of TTF2 (118–126) is shown. **(F)** TTF2 and pol ε interact via residues 120–125 of TTF2. Anti-Flag resin was optionally incubated with Flag-tagged pol ε (tagged on POLE1), incubated with TTE expressing TTF2(1–200)^WT^ or TTF2(1–200)^Δ120−125^, recovered, and eluted proteins were blotted as indicated.

To understand how TTF2 promotes CMG unloading, we used AlphaFold-Multimer (25) to address whether TTF2 and TRAIP are predicted to interact. AF-M detected no confident interaction between the full-length proteins (predictomes.org). Because performing structure prediction with segments of proteins increases the likelihood of detecting interactions (26, 27), we divided TRAIP into individual domains and folded each one with TTF2^1–200^. In this analysis, TTF2^1−200^ was predicted to interact with the C-terminal disordered region of TRAIP (Table S1, rows 6 and 9), suggesting that the ZF contacts T325. This prediction became highly confident when the proteins were further trimmed (Table S1, rows 10–11). Repeating the folding with a phosphorylated peptide in AlphaFold 3 (28) predicted that TRAIP phospho-threonine 325 interacts with residues K16 and R20 in a positively charged pocket on TTF2’s Zn finger (Fig. 3A; Table S1, rows 12–13). Consistent with this interaction being important to promote TRAIP function, TTF2(1–200)^K16A^ did not support ARP formation, CMG unloading, or CMG ubiquitylation (Fig. 3B–D, and fig. S5D). Together, these results suggest that the Zn finger domain of TTF2 docks onto phosphorylated TRAIP. Further evidence for this interaction is shown below.

### TTF2 also interacts with DNA pol ε

The results described above document a phospho-regulated interaction between TTF2 and TRAIP, but they do not explain how this complex contributes to mitotic TRAIP function. To address this, we used a high throughput pipeline using AF-M (29) to fold TTF2 with ~280 genome maintenance proteins including the 70 core DNA replication factors. Among the latter group, the most confidently predicted partner was the POLE2 subunit of DNA polymerase ε (pol ε)(Table S1, rows 14–15; predictomes.org) (29). AF-M predicted that the conserved motif adjacent to the Zn finger (residues 120–125), which is positively charged, docks into a negatively charged groove on the surface of POLE2 that is accessible in the human replisome (Fig. 3E and fig. S5F). Consistent with this prediction, DNA pol ε expressed in insect cells co-immunoprecipitated TTF2^WT^ but not TTF2^Δ120−125^, which lacks the POLE2-interaction motif (Fig. 3F). Importantly, TTF2(1–200)^Δ120−125^ did not support ARP formation, CMG unloading, or CMG ubiquitylation (Fig. 3B–D; fig. S5D). Interestingly, TTF2(1–200)^Δ120−125^ did not bind efficiently to chromatin, nor did TTF2(1–200)^K16A^ (Fig. 3C–D). We conclude that TTF2 must bind POLE2 to support TRAIP function. Moreover, if TTF2 cannot bind TRAIP, TTF2 replisome binding itself is reduced, suggesting that TRAIP and TTF2 bind replisomes cooperatively.

### A TRAIP-TTF2 chimera bypasses the requirement for S295 and T325 phosphorylation

**Fig. 4:**
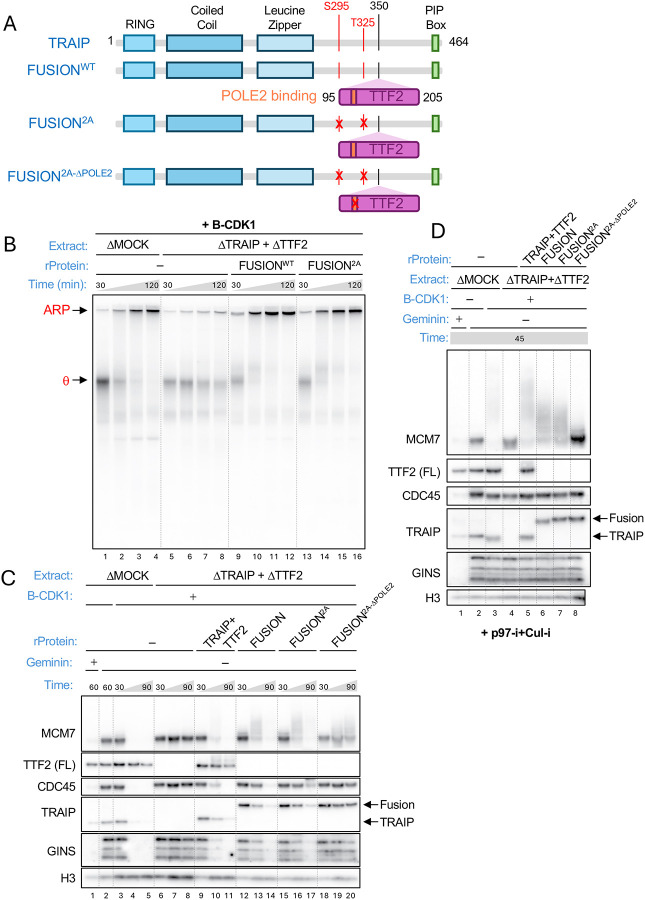
A TRAIP-TTF2 fusion bypasses the need for B-CDK1 phosphorylation. **(A)** Schematic of TRAIP-TTF2 fusions. TTF2 amino acids 95–205 were inserted between residues 350 and 351 of TRAIP. Orange box, POLE2 binding region of TTF2. Red X, mutations. **(B-D)** A TRAIP-TTF2 fusion lacking CDK sites (2A) supports ARP formation (B), CMG unloading (C), and CMG ubiquitylation (D), and a fusion also deficient in POLE2 binding (2A-ΔPOLE2) fails to support CMG unloading (C) and ubiquitylation (D). ARP, CMG unloading, and CMG ubiquitylation assays were performed as in Figs 1D, E, and F, respectively, using mock-depleted extract or extract depleted of both TRAIP and TTF2. Depleted extract was supplemented with the indicated fusion proteins or a mixture of TRAIP and TTF2.

To further test the model that B-CDK1 phosphorylation of TRAIP promotes the interaction of TRAIP and TTF2, we generated a fusion protein in which TTF2 residues 95–205, encompassing the POLE2 interaction motif, were inserted between residues 350 and 351 in TRAIP (Fig. 4A; FUSION^WT^). This chimeric construct restored ARP formation, CMG unloading, and CMG ubiquitylation in extracts depleted of TRAIP and TTF2 (Fig. 4B–D; fig. S5E). Importantly, the chimera supported ARP formation even when residues S295 and T325 were mutated to alanine (Fig. 4A–D; FUSION^2A^; fig. S5E). Thus, when TTF2 and TRAIP were covalently linked, these CDK phosphorylation sites in TRAIP were not essential. This strongly supports the conclusion that the main function of T325 phosphorylation is to facilitate TRAIP’s interaction with TTF2. In contrast, when we also mutated the POLE2 interaction motif, the chimera failed to restore CMG unloading or ubiquitylation (Fig. 4A, C–D; FUSION2^2A-ΔPOLE2^; fig. S5E). Therefore, TTF2 bridges an interaction between phosphorylated TRAIP and the POLE2 subunit of pol ε within the replisome.

### RNAPII eviction requires the TTF2 ATPase domain

**Fig. 5.**
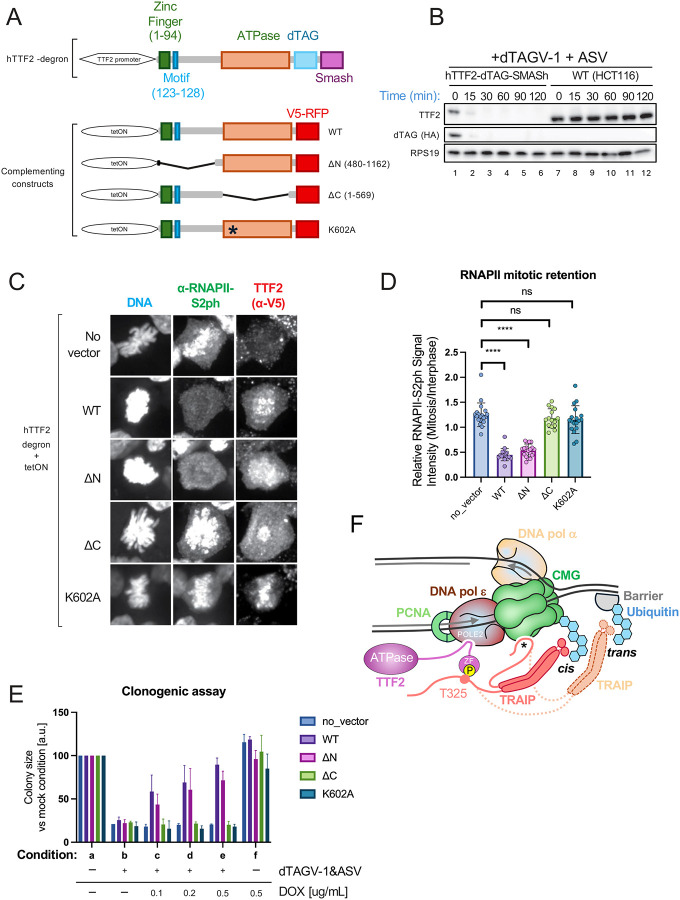
The TTF2 ATPase domain is sufficient for RNAPII eviction from mitotic chromosomes and cell proliferation. **(A)** Top, schematic of the human TTF2 gene with its encoded structural domains and the engineered C-terminal dTAG and Smash degrons. Bottom, complementing constructs under doxycycline (DOX) inducible tetON promoter. **(B)** The degron-tagged TTF2 construct is degraded within 30 minutes of ligand addition. The TTF2-degron and parental cell lines were exposed to dTAGV-1 and ASV ligands for the indicated times, and lysates were blotted for TTF2. **(C)** The TTF2 ATPase domain is required for RNAPII eviction. Immunofluorescence microscopy detection of RNAPII (S2-phosphorylated C-terminal domain) and complementing TTF2 constructs (α-V5 tag). Endogenous TTF2 was degraded, and TTF2 construct expression was induced (0.2 ug/mL DOX). Mitotic chromosome compaction was visualized using Hoechst dye and indicated with yellow arrowheads. **(D)** Quantification of RNAPII-S2ph signal intensities on mitotic chromatin relative to interphase nuclei. Cells were complemented with wild-type TTF2 or the indicated mutants after endogenous TTF2 degradation. RNAPII-S2ph intensities were measured after background subtraction and normalized to interphase levels. Data are presented as mean ± SEM; *p < 0.05. (E) The TTF2 ATPase domain is sufficient for cell viability and proliferation. Quantification of colony sizes from clonogenic assays is presented. **(F)** Model of TRAIP and TTF2 interaction with the mitotic replisome. The Zinc finger (ZF) of TTF2 binds phosphorylated T325 of TRAIP (yellow P) and a conserved motif adjacent to the ZF binds POLE2. In this manner, TTF2 forms a bridge between TRAIP and the replisome. *, putative contact point between TRAIP and the replisome (see Discussion). The flexible attachment of TRAIP to the replisome allows both cis ubiquitylation of CMG (TRAIP with solid lines) and trans ubiquitylation of a barrier ahead of the fork (TRAIP with dotted lines). TTF2 engaged in RNAPII eviction via the ATPase domain is not shown.

Finally, we asked whether TTF2 evicts mitotic replication and transcription complexes by similar or distinct mechanisms. To address this question, we modified the endogenous TTF2 locus in HCT116 colon cancer cells so that it encodes a dTAG-SMASH degron (Fig. 5A and fig. S6A), allowing TTF2 destruction within ~30 minutes after addition of the small molecules dTAGV-1 and ASV (Fig. 5B). Consistent with previous siRNA results (19), TTF2 degradation led to a failure of RNAPII eviction from mitotic chromosomes (fig. S6B). Full length TTF2 and a mutant lacking the entire N-terminus (including the ZF and adjacent POLE2 interaction site, TTF2^ΔN^) both rescued RNAPII eviction (Fig. 5A, C, and D; fig. S6C). In contrast, TTF2 carrying a point mutation in the Walker A motif that is expected to disrupt ATP hydrolysis (TTF2^K602A^) and TTF2 lacking the entire C-terminal ATPase domain (TTF2^ΔC^) were both unable to support RNAPII eviction (Fig. 5A, C, and D; fig. S6B,C), consistent with prior observations that TTF2-dependent release of transcripts from stalled RNAPII *in vitro* requires ATP (30). Expression of TTF2^WT^ and TTF2^ΔN^, but not TTF2^K602A^ or TTF2^ΔC^ supported cell viability in cells lacking endogenous TTF2 (Figure 5E, compare conditions a, b, and e; fig. S6C–E). Therefore, TTF2’s essential function correlates with its ability to displace RNAPII. Collectively, our data show that TTF2 evicts replisomes and transcription complexes by distinct mechanisms in mitosis. The removal of RNAPII appears to be a constitutive process that occurs in every cell cycle, whereas the removal of replisomes is only needed when unreplicated DNA persists into mitosis.

## DISCUSSION

How vertebrate cells respond to incomplete DNA replication remains poorly understood. Previous evidence showed that TRAIP ubiquitylates CMG helicases that remain on chromatin in mitosis, leading to replisome unloading, fork breakage, and alternative end-joining (14, 16, 17). We proposed that this pathway minimizes genome instability by generating one intact chromatid and another with a small deletion (14) , as seen during common fragile site expression (fig. S1C; (3)). In this paper, we used frog egg extracts to identify an essential role for TTF2 in supporting the mitotic function of TRAIP (Fig. 5F). We show that in mitotic extracts, TRAIP is phosphorylated on T325, and our evidence suggests that this modification mediates binding to the ZF of TTF2, although we have not shown the interaction directly. We additionally find that a short, conserved TTF2 motif adjacent to the ZF mediates TTF2 binding to the POLE2 subunit of pol ε. An N-terminal domain of TTF2 that includes the ZF and adjacent motif is sufficient to support TRAIP function, whereas TTF2’s ATPase domain is dispensable. Collectively, these results suggest that TTF2 forms a phospho-dependent bridge between TRAIP and the replisome that allows TRAIP to strip all replisomes from mitotic chromosomes. Furthermore, while TTF2 also strips transcription complexes from mitotic chromosomes, this process involves its ATPase activity.

We previously showed that TRAIP is highly constrained in interphase, being limited to ubiquitylation of protein barriers encountered by the fork *in trans*, whereas in mitosis it can also ubiquitylate the CMG with which it associates *in cis*. Importantly, TRAIP^S295^, TRAIP^T325^, and TTF2 are required for the mitotic but not the interphase functions of TRAIP. Therefore, a fundamental re-organization must take place in mitosis, whereby interactions that constrain TRAIP’s RING domain in interphase are replaced with or supplemented by a less constrained mitotic binding mode. The mitotic configuration of the replisome requires regions of TRAIP and TTF2 that are both predicted to be disordered. We therefore propose that TRAIP is flexibly associated with the replisome in mitosis, giving its RING domain freedom to ubiquitylate both the replisome and proteins that block its path (Fig. 5F).

Our observations indicate that TTF2 likely does not recruit TRAIP through a simple, linear pathway. We found that TTF2 mutations predicted to disrupt TRAIP binding (K16A and Δ120–125) also prevent efficient TTF2 chromatin binding. This suggests that TRAIP and TTF2 bind replisomes cooperatively, with the two factors not only interacting with each other, but both also making independent contacts to the replisome. While TTF2 clearly contacts POLE2, a direct TRAIP-replisome contact remains to be identified. Interestingly, when we deplete TTF2, levels of chromatin-associated TRAIP are unaffected (Fig. 3C, lanes 2 and 5). We speculate that this is because two dimers of TRAIP are associated with the replisome in mitosis, including one that is bound in the interphase mode that is not affected by loss of the functional interaction with TTF2.

Previous biochemical experiments showed that TTF2-dependent transcript release from stalled RNAPII requires ATP (30). Consistent with this finding, we found that RNAPII eviction from mitotic chromosomes in cells is inhibited by a Walker A mutation in the TTF2 ATPase domain, suggesting ATPase activity is required. Moreover, the C-terminal ATPase domain is sufficient to support RNAPII eviction. Therefore, the segments of TTF2 required for replisome and RNAPII eviction are non-overlapping, arguing that TTF2 stimulates these two processes by distinct mechanisms. Whether there are situations, such as replisome encounter with transcription complexes in mitosis, where the two halves of TTF2 act in concert, is an interesting question for future investigation.

In conclusion, we identify TTF2 as a new component in the response to unreplicated DNA in mitosis. The specific requirement for TTF2 in mitotic TRAIP function implies that the replisome undergoes major structural changes to meet cell-cycle specific challenges to genome integrity.

## Materials and Methods

### Recombinant protein expression

To purify biotinylated LacR, a plasmid expressing avidin tagged LacR pET11a[LacR-Avi] and a plasmid expressing biotin ligase pBirAcm (Avidity, Denver, CO) were co-transformed into T7 Express cells (New England Biolabs). Cultures were grown in LB media containing 50 mM biotin at 37°C (Research Organics, Cleveland, OH). Co-expression of LacR-Avi and the biotin ligase was induced by addition of 1mM IPTG (Isopropyl β-D-thiogalactoside, Sigma, St. Louis, MO). Biotinylated LacR-Avi was then purified as described (31), concentrated, flash frozen and stored at −80°C.

To purify DNA polymerase ε complex (pol ε), the 4 genes encoding Xenopus laevis POLE, POLE2, POLE3, and POLE4 were cloned individually into pLIB vectors using Gibson Assembly. POLE was cloned with a 3xFLAG tag on the N-terminus and a 10xHis tag on the C-terminus while POLE4 was cloned with a Calmodulin tag at the C-terminus. The pLIB vectors were then used to assemble a pBIG1-a baculovirus expression vector encoding all 4 subunits following the biGBac method (32). This vector was transformed into DH10Bac cells to generate the bacmid, and Sf9 cells were transfected with the bacmid to produce baculovirus that was amplified 3 times. The amplified baculovirus was used to transfect 1L of Sf9 cells. After 72hrs, cells were collected, washed with 1xPBS, flash frozen in liquid nitrogen and stored at −80°C. For pol ε purification, cell pellets were lysed in lysis buffer consisting of buffer H (25 mM Hepes-KOH pH=7.6, 500 mM KOAc, 10% glycerol, 1 mM DTT, 0.05% Igepal), 1 mM PMSF and 2 tablets cOmplete protease inhibitor (Roche #5056489001) per 50 mL of lysate. DNA was sheared enzymatically by benzonase and mechanically by sonication (1s ON 5s OFF for 1 min at 45% amplitude). The lysate was clarified by centrifugation at 35,000 rpm at 4°C for 1hr. Cleared lysate was allowed to bind anti-FLAG M2 affinity gel (Sigma-Aldrich #A2220–10ML) for 2hr at 4°C. The resin was equilibrated and washed extensively with lysis buffer, then the protein was eluted with buffer containing lysis buffer + 0.15mg/mL 3xFLAG peptide (Sigma-Aldrich #F4799–25MG). 2mL of Ni-NTA superflow resin (Qiagen #30430) was equilibrated with Buffer A (Buffer H + 20 mM Imidazole). Then 20 mM Imidazole was added to the eluted protein, which was then bound to the resin for 2hr at 4°C. The resin was washed extensively with Buffer A, and the protein was eluted with Buffer B (Buffer H+ 200 mM Imidazole). The eluted protein was concentrated using a 100 kDa MWCO Amicon centrifugal unit and loaded onto a superose 6 Increase 10/300 GL (Cytiva) column equilibrated with sizing buffer (Buffer H without Igepal). The protein was eluted isocratically, concentrated, flash frozen and stored at −80°C.

To purify TTF2 (1–200), the first 200 codons of Xenopus laevis TTF2 were cloned with N-terminal GST-3C tag into a plasmid and expressed in E.coli (GST-3C-TTF2-N). Recombinant TTF2(1–200) protein was purified on a glutathione-sepharose column; GST was cleaved with HRV 3C protease and TTF2(1–200) was further purified by size exclusion chromatography, concentrated, flash frozen and stored at −80°C. Commercially available Cyclin B1-CDK1 was obtained from EMD Millipore (Cat #14–450M).

### Expression of proteins in transcription-translation extract (TTE)

TRAIP, TTF2, and FUSION (various constructs) were cloned into pF3A WG (BYDV) Flexi vectors. Mutations were introduced using a Q5 Site-Directed Mutagenesis Kit (NEB #E0554S). Plasmids were amplified in DH5α cells and purified using QIAprep Spin Miniprep Kits (Qiagen). Residual contaminants were further removed from plasmid preparations using AMPure XP Reagent (Beckman Coulter #A63881), and eluted with 10mM, Tris pH 8.0. To express recombinant proteins, two volumes of 100 ng/μL TRAIP expression plasmid and 150 ng/μL of TTF2 or Fusion plasmid were mixed with three volumes of TnT^®^ SP6 High-Yield Wheat Germ Protein Expression System (Promega # L326A) and incubated at 25°C for 2 hours. Extracts containing expressed proteins were aliquoted, flash frozen, and stored at −80°C.

### Plasmid replication substrates

The 4.6 kb LacR plasmid containing an array of 48 lacO (48xLacO) was previously described (31). A plasmid containing a site-specific cisplatin-ICL (pICLPt) was constructed as previously described (33). Briefly, parental plasmid linearized with BbsI was ligated with purified cisplatin oligonucleotide duplexes comprising Pt_Top and PT_Bottom, and the resulting supercoiled plasmid was purified using a cesium chloride gradient.

### Xenopus egg extracts and in vitro DNA replication

Experiments involving adult female (Nasco #LM0053MX) Xenopus laevis performed at Harvard Medical School were approved by the Harvard Medical Area Standing Committee on Animals. The institution has an approved Animal Welfare Assurance (D16–00270) from the NIH Office of Laboratory Animal Welfare. Xenopus egg extracts were prepared as previously described (34).

All DNA replication reactions were performed at 22°C. To replicate pICL in interphase egg extracts (Fig. S3), 1 volume of 75 ng DNA/μL plasmid DNA was added into 9 volumes of HSS and incubated for 30 minutes to promote licensing (ORC-dependent loading of MCM2–7 double hexamers). Subsequently, 1 volume HSS-DNA was mixed with 2 volumes of 50% NPE to initiate CDK2-dependent replication. To induce replication fork stalling using the LacR barrier in mitosis, one volume of 48XLacO (150 ng/μL) was incubated with one volume of 50 μM recombinant LacR for 1 hour. Next, 2 volumes of LacR-DNA were mixed with 8 volumes of HSS to allow licensing. Subsequently, 1 volume of HSS-DNA-LacR was mixed with 2 volumes of 50% NPE to initiate CDK2-dependent replication. For mitotic replication, fifteen minutes after initiation, 0.5 volumes of recombinant B-CDK1 (1 μg/μL) was added to 14.5 volumes of the replication reaction, resulting in a final concentration of 33.3 ng/μL B-CDK1. As indicated, p97 inhibitor (p97-i) NMS-873 (Sigma Cat #SML1128–5MG) and Cullin inhibitor (Cul-i) MLN-4924 (Active Biochem Cat #A-1139) were added to replication reactions 10 minutes after NPE addition at a final concentration of 250μM.

### Analysis of DNA replication and repair intermediates

To monitor total DNA synthesis and repair intermediates of ICL and stalled replication forks in mitosis, HSS-DNA mixtures were supplemented with 0.16 μCi/μL of [α−^32^P]dATP(Perkin Elmer #BLU512H500UC) just before the addition of NPE. At the indicated times after initiating replication by NPE addition, 0.8ul of the replication reactions were stopped in 9 volumes of replication stop buffer (80 mM Tris-HCl pH 8.0, 8 mM EDTA, 0.13% phosphoric acid, 10% Ficoll 400, 5% SDS, 0.2% bromophenol blue) and supplemented with 20 μg of proteinase K (Roche #3115879001). The samples were incubated at 37°C for 1 hour to digest proteins. Samples were separated on 0.9% native agarose gels and 1X TBE buffer (89 mM Tris, 89 mM Boric acid, 2 mM EDTA pH 8.0). The gels were sandwiched between positively charged nylon membrane (Roche #11417240001) to prevent loss of nucleic acids, and paper towels to extract water, for 1 hour. Subsequently, gels were dried at 80°C under vacuum, exposed to phospho screens, and imaged on the Typhoon FLA 700 PhosphorImager (GE Healthcare).

### Immunodepletions and rescue experiments

For immunodepletion of endogenous TRAIP, anti-TRAIP antibodies (Biosynth project #3472), affinity-purified as previously described (10) were used. Three volumes of the 1 mg/mL antibodies were incubated with two volumes of magnetic Protein A beads (Dynabeads M-280, Invitrogen #10001D) by gently rotating at 4°C overnight. Three volumes of 100% HSS or 55% NPE were immunodepleted by three rounds of incubation with two volumes of antibody-immobilized magnetic Protein A beads, by gently rotating at 4°C for 1 hour per round.

For immunodepletion of endogenous TTF2, a rabbit polyclonal antibody against recombinant TTF2(1–200) (Pocono R37741) was raised. Three volumes of 1 mg/mL affinity-purified antibody were incubated with 2 volumes of magnetic Protein A beads by gently rotating at 4°C overnight. 3 volumes of 100% HSS were immunodepleted by four rounds of incubation with 2 volumes of antibody-immobilized magnetic Protein A beads or 55% NPE was immunodepleted by three rounds of incubation with 2 volumes of antibody-immobilized magnetic Protein A beads, by gently rotating at 4°C for 1 hour per round.

For rescue experiments using TRAIP expressed in TTE, TTE was added to TRAIP-depleted NPEs at a final concentration of 6%, and the mixture was incubated for 5 minutes at room temperature before initiating replication. For rescue experiments using TTF2 expressed in TTE, TTE was added at a final concentration of 10% to NPE, and the mixture was incubated for 5 minutes at room temperature before initiating replication.

### Plasmid pull-down

Plasmid pull-downs were performed as described (35) with minor modifications. Briefly, 0.2 μM of biotinylated recombinant LacR proteins were immobilized on one volume of streptavidin-coated magnetic beads (Dynabeads M-280, Invitrogen 11206D) in six volumes of binding buffer (50 mM Tris-HCl pH 7.5, 150 mM NaCl, 1 mM EDTA, 0.02% Tween 20) for 1 hour at room temperature. The beads were washed extensively with stop buffer (20 mM HEPES-KOH pH 7.7, 50mM KCl, 5 mM MgCl2, 0.5 M sucrose, 0.25 mg/mL BSA, 0.03% Tween 20), and resuspended in six volumes of the same buffer, and chilled on ice. At the indicated time points beads were aliquoted and one volume of replication reaction sample was mixed with nine volumes of the bead aliquots and gently rotated for 30 minutes at 4°C. The beads were then washed tree times with the wash buffer (20 mM HEPES-KOH pH 7.7, 50 mM KCl, 5 mM MgCl2, 0.25 mg/mL BSA, 0.3% Tween 20). Plasmid bound proteins were eluted from the beads by boiling with 1X Laemmli buffer and subjected to analysis by SDS-PAGE and immunoblotting or mass spectrometry analysis. For immunoblotting, proteins eluted from 10 ng of plasmid were loaded in each well.

### Immunoprecipitation of TTF2 and TRAIP by recombinant pol ε

For the immunoprecipitation of TTF2(1–200) (Fig. 3F), WT TTF2(1–200) and the indicated mutants were expressed in TTE (as described above). Purified recombinant FLAG-tagged pol ε (purified as described above) was added at 1 μM to the TTE and incubated for 15 minutes at room temperature. Pierce^™^ Anti-DYKDDDDK (anti-FLAG peptide antibody) Magnetic Agarose beads (Thermo Scientific #A36797) were washed thrice in IP wash buffer (10 mM HEPES-KOH pH 7.7, 50 mM KCl, 2.5 mM MgCl2, 250 mM sucrose, 0.02% Tween 20) and used in aliquots containing 0.5 μL of packed beads. Each aliquot of beads was incubated with 15 μL of the respective pol ε mixture for 1 hour at 4°C with gentle rotation. The beads were then washed thrice with cold IP wash buffer. To elute bound proteins, each aliquot of beads was incubated with 15 μL of IP wash buffer containing 1 mg/mL 3x FLAG peptide (Sigma-Aldrich #F4799) for 1 hour at room temperature with gentle rotation. The eluates were mixed with an equal amount of 2x Laemmli buffer and boiled. 6 μL of each sample was loaded on each gel and analyzed by immunoblotting.

### SDS-PAGE and immunoblotting of samples from Xenopus egg extract experiments

All replication samples were quenched and boiled for 2 minutes at 95°C in Laemmli buffer (50 mM Tris-HCl pH 6.8, 2% SDS, 10% glycerol, 0.1% bromophenol blue, 5% β-mercaptoethanol). Except for Fig 1B (see below), samples were run on 4–15% Criterion TGX Precast Midi Protein Gels (Bio-Rad #5671085) using Tris-Glycine-SDS Running Buffer (25 mM Tris-HCl pH 8.3, 192 mM glycine, 0.1 % SDS). To resolve phosphorylated TRAIP in Fig. 1B, PhosTag reagent (Wako #AAL-107) containing SDS-PAGE gels was used (36) with the following minor modifications. The resolving gels were composed of 6% acrylamide:bisacrylamide (29:1), 0.5% agarose, 375 mM Tris pH 8.8, 0.1% SDS, 0.001% TEMED, 30 μM ZnCl2, 0.05% APS, 30 μM Phos-tag acrylamide. The stacking gels were composed of 3% acrylamide: bisacrylamide (29:1), 125 mM Tris pH 6.8, 0.001% TEMED, 0.05% APS. Except in the case of TTF2(1–200), gels were transferred to 0.45 μM PVDF membranes (Thermo Scientific #88518) in transfer buffer (25 mM Tris pH 8.5, 192 mM glycine, 20% methanol) at 300mA for 1 hour. Gels for TTF2(1–200) were transferred to 0.22 μM Nitrocellulose (GE Healthcare #10600001) membrane as described above. For Fig. 1C, the membranes were blocked with 5% (w/v) non-fat milk prepared in 1X PBST for 1 hour at room temperature by gentle agitation and incubated with primary antibodies diluted in 1XPBST containing 1% (w/v) BSA overnight at 4°C with gentle agitation. Membranes were then washed 3 times for 10 minutes with 1XPBST and incubated with secondary antibodies diluted in 5% (w/v) non-fat milk prepared in 1x PBST for 1 hour at room temperature with gentle agitation. Membranes were then washed 3 times for 10 minutes with 1XPBST and imaged using an Amersham Imager 600 (GE Healthcare). To immunoblot for phospho-specific Traip S295 and T325 antibodies in Fig. 1C, the protocol above was used except that 1X TBST and %5BSA were used instead of 1XPBST and non-fat milk at the indicated steps. Rabbit polyclonal antibodies against phosphorylated Xenopus Traip S295 (Biosynth #G5978) and T325 (Biosynth #G5979) were raised by immunizing with the corresponding phosphorylated short peptides and were affinity-purified. Antibodies further precleared with B-CDK1 supplemented TRAIP depleted HSS.

For western blotting of Xenopus proteins, the following rabbit polyclonal antibodies were used as primary antibodies, at the specified dilutions:

TRAIP (1:10,000) (10)

TRAIP ps295 (1:20000, described above)

TRAIP pT325 (1:12500, described above)

MCM7 (1:12,000) (37)

CDC45 (1:10,000) (37)

GINS (1:5,000) (38)

TTF2 (1:2500, described above)

Histone H3 (1:500, Cell Signaling #9715, RRID: AB_331563).

The goat anti-rabbit horseradish peroxidase-conjugated secondary antibodies (Jackson ImmunoResearch, 111-035-003, RRID: AB_2313567) were used at 1:20,000 dilution for MCM7, POLE, POLE2, POLE3, POLE4 GINS and H3 blotting.

The Light chain-specific mouse anti-rabbit horseradish peroxidase-conjugated secondary antibodies (Jackson ImmunoResearch, 211-032-171, RRID: AB_2339149) were used at 1:10,000 dilution for TRAIP, TTF2, and CDC45 blotting.

### MS sample preparation

For plasmid pull down, 8 μl of the total reaction was withdrawn at the indicated time points and plasmids and associated proteins were recovered by plasmid pull down using LacI coated beads as described above with minor modifications. The samples were washed twice with 20 mM HEPES-KOH (pH 7.7), 100 mM KCl, 5 mM MgCl2, 0.25 mg ml−1 BSA, and 0.03% Tween-20 and once with with 50 ml ELB (10 mM HEPES/KOH at pH7.7, 50 mM KCl, 2.5 mM MgCl2) to remove residual detergent. The beads were diluted with ABC (50 mM ammoniumbicarbonate) and digested with 2.5 mg trypsin (Sigma) and 2M Urea and incubated for 16 hours at 30 °C. Supernatant was cleared by spinning through 0.45um ultrafree filters before stopping the digestion with Trifluoroacetic acid. NaCl was added to 400 mM final concentration and peptides were acidified and purified by stage tipping on C18 material(39).

### LC-MS/MS analysis

Peptides were separated on reversed phase columns (50 cm, 75 μm inner diameter, packed in-house with ReproSil-Pur C18-AQ 1.8 μm resin (Dr. Maisch GmbH) and directly injected into a quadrupol orbitrap mass spectrometer (Q Exactive HF, Thermo Scientific, Germany). Using a nanoflow HPLC (Thermo Scientific, Odensee), peptides were loaded in buffer A (0.5% acetic acid) and eluted with a three hour non-linear gradient from 5–95% buffer B (80% acetonitrile, 0.5% acetic acid) at a constant flow rate of 250 nl/min. For the reversed phase separation, the column was maintained at a constant temperature of 60°C. The mass spectrometer was operated in a data dependent fashion using a top 15 method for peptide sequencing.

### MS Data processing

Raw data were analyzed with the MaxQuant software (version 2.0.1.0) (40). A false discovery rate (FDR) of 0.01 for proteins and peptides and a minimum peptide length of 7 amino acids were required. MS/MS spectra were searched against a non-redundant Xenopus database (see (41) for details). For the Andromeda search, trypsin allowing for cleavage N-terminal to proline was chosen as enzyme specificity. Cysteine carbamidomethylation was selected as a fixed modification, while protein N-terminal acetylation and methionine oxidation were selected as variable modifications. Maximally two missed cleavages were allowed. Protein identification required one unique peptide to the protein group. Raw intensities were normalized using the label free quantification (LFQ) algorithm implemented in MaxQuant. Match between run was enabled to transfer identities between runs of the same replicate group.

### Statistical analysis and visualization of MS data

Protein groups were filtered to eliminate contaminants, reverse hits, and proteins identified by site only. For the heat map (Fig. 2A and fig. S4B), LFQ intensities were log2 transformed and for each protein z-scores were calculated across all conditions. To visualize heatmaps in a more compressed form, z-scores of individual subunits were averaged for selected complexes (see Suppl. table 2 for the z-scores of all proteins). Proteins were manually annotated and sorted according to their function in DNA replication and DNA repair. To identify proteins with significant abundance changes between the four conditions, LFQ intensities were log2 transformed, and missing values were imputed with random values drawn from a normal distribution centered around the detection limit of the MS instrument (Perseus imputation; width=0.3, down shift = 1.8). Two sample Student’s t-tests with permutation-based FDR control were carried out in Perseus. For these tests, three valid values in at least one triplicate of either of the tested conditions was required. FDR was adjusted for multiple testing by the Benjamini-Hochberg procedure using a significance threshold of FDR<0.05 (see suppl. table 2). Data visualization was carried out in R. All scripts are available upon request.

### Cell lines and Constructs

The HCT116 cell line and its derivatives were grown in McCoy’s 5A medium (30–2007, ATCC) supplemented with 10% tetracycline-free FBS and 1% penicillin-streptomycin mixture in 5% CO2 at 37°C. TTF2-degron cell lines were created using CRISPR Cas9 genome editing with marker-free co-selection (42). To this end, HCT116 cells were transfected using Neon Transfection System with a mixture of three DNA vectors: (1) double Cas9-gRNA vector (containing Cas9 and gRNA against TTF2 STOP codon and ATP1A1 ouabain suppression mutation site; eSpCas9(1.1)_gATP1A1_gT2C2), (2) TTF2 degron HDR targeting template (hTTF2_CT_dTAG_SMASh2), and (3) ATP1A1 ouabain resistance HDR targeting template (ATP1A1_Q11RN129D). Transfected cells were selected for growth in ouabain (10 uM; O3125, Sigma) containing media. Resistant cells were FACS sorted to single cells in 96-well plates, and clones were screened by PCR for degron cassette integration and by immunoblotting for TTF2 depletion under degron activation. TTF2-degron depletion was induced by supplementing media with 0.5 uM dTAGV-1 (6914, Tocris)(43) and 1 uM ASV (HY-14434, MedChemExpress)(44).

To complement the TTF2-degron cell lines with ectopically expressed doxycycline (DOX) inducible TTF2 versions, the human TTF2 open reading frame was cloned into a pLIX403 lentiviral vector under tetON promoter with C-terminal V5 affinity tag and RFP fluorescent protein tag. The resulting pLIX403_hTTF2-V5-RFP was mutagenized by PCR and NEBuilder HiFi assembly kit cloning to obtain TTF2 mutant constructs. pLIX403 based vectors were then used to produce lentivirus. Lentivirus-infected TTF2-degron cell lines were selected on puromycin, FACS sorted for RFP expression (under transient DOX treatment), and single-cell cloned to obtain clonal cell lines. TTF2 ectopic expression was induced by addition of 0.1–0.5 ug/mL DOX in the media for 24 hours or longer.

### Immunofluorescence and Microscopy

Cells were seeded on poly-D-lysine coverslips in 12-well plates at a concentration of 50 k/mL (day 1). On day 4, the coverslips with cells were washed with PBS and fixed with simultaneous permeabilization using PTEMF solution (20 mM PIPES pH 6.8, 1 mM MgCl2, 10 mM EGTA, 0.2 % Triton X-100, 4 % paraformaldehyde) at RT for 15 min. Coverslips were washed thrice with PBS (5 min each wash here and after) and kept at 4 °C until staining (up to a week). For immunostaining, fixed coverslips were blocked with blocking buffer (3 % BSA in PBS) for 1 hour at RT, incubated with primary antibodies diluted in blocking buffer for 1 hour at RT, washed thrice with Wash buffer (0.05 % Triton X-100 in PBS), and incubated for 1 hour at RT with secondary antibodies diluted in blocking buffer. After removing secondary antibodies and three more washes with Wash buffer, the coverslips were incubated with 1:2500 dilution of Hoechst stain (H3570, Invitrogen) for 10 min at RT. Hoechst solution was removed, and coverslips were washed thrice with PBS. Coverslips were mounted in Prolong Diamond Antifade (P36965, Invitrogen) on imaging slides, cxured overnight at RT and imaged using a spinning disc confocal microscope (Nikon) with a 60X objective. Images were processed in Fiji. Z-stacks were max intensity projected. Primary antibodies: α-RNAPII-S2ph 1:400 (ab5095, rabbit, Abcam)), α-V5 1:200 (R960–25, mouse, Thermo), α-TTF2 1:100 (sc-514996, mouse, SantaCruz BioTechnology). Secondary antibodies: goat anti-rabbit Alexa Fluor 488 1:1000 (A32731, Invitrogen), goat anti-mouse Alexa Fluor 568 1:1000 (A11031, Invitrogen).

### Clonogenic survival assay

Cells were seeded in 6-well plates at 200, 400, 600 (Fig. S6D), or 450 cells per well (Fig. 5D) (day 1). Where indicated, doxycycline (DOX) was added on day 2 and degron ligands (dTAGV-1 and ASV) were added on day 3. Medium and drugs were refreshed every 3 days. On day 14, media was aspirated, the wells were washed with PBS, colonies were fixed by methanol for 20 min at RT and stained by crystal violet solution (0.4 % (w/v) crystal violet, 20 % methanol)) for 1 hour at RT. The crystal violet solution was then recovered, and wells washed thrice with warm tap water. The plates were dried overnight and photographed with iPhone SE; images were cropped in ImageJ.

### Immunofluorescence microscopy image quantification

The immunofluorescence microscopy images were analyzed using custom Fiji macro and Excel. In Fiji, image hyperstacks were first collapsed (Image > Stacks > Z project > MAX intensity option), and individual channels set to grays (LUT > Grays). To measure signal intensity for each channel on and outside of chromatin, a nuclear (DNA) mask was first created. To this end, the DNA channel (Hoechst) image was duplicated, converted to 8-bit (Image > Type > 8-bit), smoothed by applying Median filter (with 3 pixels setting) and thresholded with ‘Huang’ method (Image > Adjust > Threshold (Huang)). Holes were filled (Process > Binary > Fill Holes), chromosome groups were separated using watershed (Process > Binary > Watershed) and obtained DNA mask was saved. Chromosome groups (interphase nuclei and mitotic chromosomes) were detected on the mask by using Analyze Particles method (with size set to 1000-Infinity). Each channel signal intensity was then measured for all chromatin groups Regions of Interest detected with the mask (ROI Manager > measure). To obtain the signal intensity outside of chromatin (background), the DNA mask was first inverted (Edit > Invert), total region outside of chromatin selected (Edit > Selection > Create Selection) and used for quantification in ROI Manager (Analyze > Tools > ROI Manager + Add > measure). Obtained measurements were imported to Excel, where RNAPII-S2ph background intensity was subtracted from intensity on chromatin. The RNAPII-S2ph signal intensity on TTF2-positive (or negative, for no_vector control) mitotic chromatin was divided by average signal on interphase nuclei of the same field of view, and obtained values were reported on the figure graph using Prism. Prism Mann-Whitney U test was used to test for statistical significance of the difference in value distributions.

### Clonogenic assay image quantification

Clonogenic assay plate images were analyzed in ImageJ and Excel. Each plate image was converted to 8-bit grayscale (Image > Type > 8-bit), and thresholded by limiting contrast to 0–150 (Image > Adjust > Threshold 0–150). To separate colonies, the watershed method was then applied (Process > Binary > Watershed). Wells were selected with the circle select tool of equal size per plate, and colonies were detected and measured using the Analyze Particles tool. Average colony size in percents was calculated in Excel by dividing the average colony size in each well by that of the mock-treated well and multiplication by 100. Average colony size values were then reported on the figure graph using Prism.

### Immunoblotting of cell-based samples

Cells were trypsinized, pelleted, washed with PBS, re-suspended in 2x Laemmli buffer and boiled for 5 minutes at 95°C. Denatured samples were resolved on 4–15% Tris-Glycine SDS PAGE, transferred to PVDF membrane, blocked with milk, washed thrice with PBS, and incubated with primary antibody overnight at 4°C with shaking. Primary antibody was decanted, membrane was washed thrice with PBS and incubated with secondary antibody for 45 minutes at RT with shaking. After three washes, the membrane was incubated with SuperSignal ECL (Thermo) and imaged with Amersham Imager. Images were cropped in ImageJ. Primary antibodies: α-TTF2 1:5,000 (PA5–96789, rabbit, Invitrogen), α-HA 1:1,000 (C29F4, rabbit, Cell Signaling), α-RPS19 1:1,000 (A304–002A, rabbit, Bethyl), α-V5 1:5,000 (R960–25, mouse, Thermo). Secondary antibodies: goat anti-rabbit HRP 1:30,000, rabbit anti-mouse HRP 1:2,000.

## Supplementary Material

Supplement 1

Supplement 2

1 Figure S1: Models for TRAIP replisome binding in the S and M phases
**(A)** Interphase configuration of TRAIP on the replisome. The TRAIP dimer binds the replisome via an unknown mechanism (?) such that the catalytic RING domains (yellow circles) are constrained and can only ubiquitylate proteins ahead of the replisome in *trans* (“Hood ornament” model). In this configuration, TRAIP ubiquitylates proteinaceous barriers ahead of the fork such as covalent DNA protein cross-links (DPCs) or a CMG that resides on the other side of a DNA interstrand cross-link (not depicted), but it cannot ubiquitylate the CMG with which it travels. **(B)** Mitotic configuration. TRAIP is flexibly attached to the replisome via an unknown mechanism (?). In this state, the catalytic RING domain is not constrained and can also ubiquitylate the hosting CMG in “cis.” **(C)** Model for common fragile site (CFS) expression. If replisomes cannot copy a difficult to replicate locus (CFS) before cells enter mitosis, the stalled CMGs undergo TRAIP-dependent ubiquitylation and p97-dependent unloading, which deprotects the forks and induces symmetric fork cleavage. The broken chromosomes (red and blue) are ligated to each other by pol θ-mediated end-joining, which leads to a small deletion with microhomology (MH) at the breakpoint and sister chromatid exchange, as seen during common fragile site expression.
Figure S2: Evidence for CDK phosphorylation of TRAIP.
**(A)** Sequence alignment of vertebrate TRAIPs. The three conserved CDK sites are indicated. **(B)** Extracts used for the experiment in Fig. 1D were blotted for TRAIP. **(C)** Analogous to (B), but for Fig. 1E&F.
Figure S3: TRAIP CDK sites are not required for interphase TRAIP function.
**(A)** A plasmid containing a cisplatin DNA inter-strand crosslink was replicated in the indicated interphase egg extracts containing [α−^32^P]dATP. At 15, 30, 60, 90 and 120 minutes, samples were separated on a native agarose gel and visualized by autoradiography. After replication initiation, forks converge on the ICL, generating a “Figure 8” structure. Upon CMG ubiquitylation by TRAIP and unloading by p97, Figure 8 is converted to a more rapidly migrating “Fast Figure 8,” probably due to re-annaealing of the ssDNA liberated upon CMG unloading and acquisition of catenanes (10, 45). Thus, conversion of Figure 8 to Fast Figure 8 is a read-out of CMG ubiquitylation. As expected, TRAIP depletion blocked Figure 8 conversion to Fast Figure 8 (lanes 7–12). Importantly, all TRAIP constructs, including ones with mutated CDK sites, restored Fast Figure 8 formation (lanes 13–36). These results show that S295 and T325 phosphorylation are not required for TRAIP-dependent CMG ubiquitylation and unloading in interphase. Irrelevant parts of the gel were removed between lanes 30 and 31.
Figure S4: Mass spectrometry analysis of proteins associated with mitotic replisomes
**(A)** LacR plasmid was replicated in the indicated egg extracts. Samples were recovered 45 and 55 minutes after NPE was added to initiate replication and either blotted for the indicated proteins or processed for mass spectrometry (B). **(B)** Heatmap of genome maintenance protein abundance in the chromatin samples from (A)(plus two additional replicates) determined by label-free mass spectrometry. Protein levels are shown as the mean of the Z scored log2 abundance in the different conditions. Full results are reported in Table S2. **(C)** Volcano plot of protein abundance in (B) in the presence and absence of p97-i (both containing B-CDK1; 45 min time point). The plot displays the mean difference of the protein intensity (−log_2_) versus the p value (−log_10_) calculated by a modified, two-sided t test. **(D)** Same as (C), except the volcano plot compares recruitment in the presence and absence of B-CDK1 (both conditions contain p97-i; 45 min time point).
Figure S5: TTF2 antibody characterization and expression of recombinant TTF2 and fusion constructs
**(A)** High speed supernatant (HSS) of egg lysate was mock-depleted, depleted of TTF2, or depleted of TTF2 and supplemented with recombinant TTF2 expressed in TTE. Extracts were blotted with an antibody raised against Xenopus TTF2. (**B)** Extracts used for Fig. 2D–F. NPE was mock-depleted or depleted of TTF2, and transcription-translation extract (TTE) programmed with empty vector or the indicated TTF2 constructs was added. The NPE-TTE mixture was blotted for TTF2. **(C)** Similar to (B), but related to Fig. 2G. **(D)** Similar to (B), but related to Fig. 3B–D. TTF2(1–200)^ΔZF^ is barely detectable, which could be due to poor expression or poor reactivity with the TTF2 antibody, which is raised against the N-terminal domain. The conclusion that that the TTF2 ZF is required for mitotic TRAIP function is clearly supported by the analysis of TTF2(1–200)^K16A^. **(E)** Similar to (B), but related Fig. 4B–D. **(F)** TTF2 binding to POLE2 does not sterically clash with other known proteins in the replisome. The predicted POLE2-TTF2 complex from Fig. 3E was aligned on POLE2 in the replisome structure (PDB: 7PLO).
**Figure S6. The TTF2 ATPase domain is sufficient for RNAPII eviction from mitotic chromosomes and cell proliferation (A)** The STOP codon of the human TTF2 gene was replaced with a degron tag sequence (dTAG-SMASh) by CRISPR Cas9 genome editing (left panel). Positive clones were identified by PCR (right panel), immunoblotting, and confirmed by immunofluorescence (B) and clonogenic assays (D). **(B)** TTF2 degradation leads to phosphorylated RNAPII retention on chromosomes in mitosis. Mitotic cells are indicated with yellow arrowheads. **(C)** Immunoblot detection of the depletion of endogenous TTF2-degron (green arrowhead) and concomitant induction of ectopic tetON complementation construct expression (orange arrowheads). **(D)** Colony formation defect of the TTF2-degron cell line compared to its unedited parent (HCT116) after exposure to the degradation-inducing ligands dTAGV-1 and ASV. **(E)** Clonogenic survival assay under conditions of endogenous TTF2 degradation and complementation with DOX-inducible ectopic TTF2 constructs. Representative colony image used in the quantification of Fig 5E.

## Data Availability

All data except MS raw files are available in the main text or the supplementary materials. MS raw files and MaxQuant output tables are available at: https://www.ebi.ac.uk/pride/ CHROMASS data set: PXD055299 Username: reviewer_pxd055299@ebi.ac.uk Password: J8C21quWiDHH
