# Supplementary figures and images for: TTF2 promotes replisome eviction from stalled forks in mitosis
Source: bioRxiv. 2024 Nov 30:2024.11.30.626186. Preprint. [Version 1] doi: 10.1101/2024.11.30.626186 (PMC11623681; doi:10.1101/2024.11.30.626186)

Figure S1

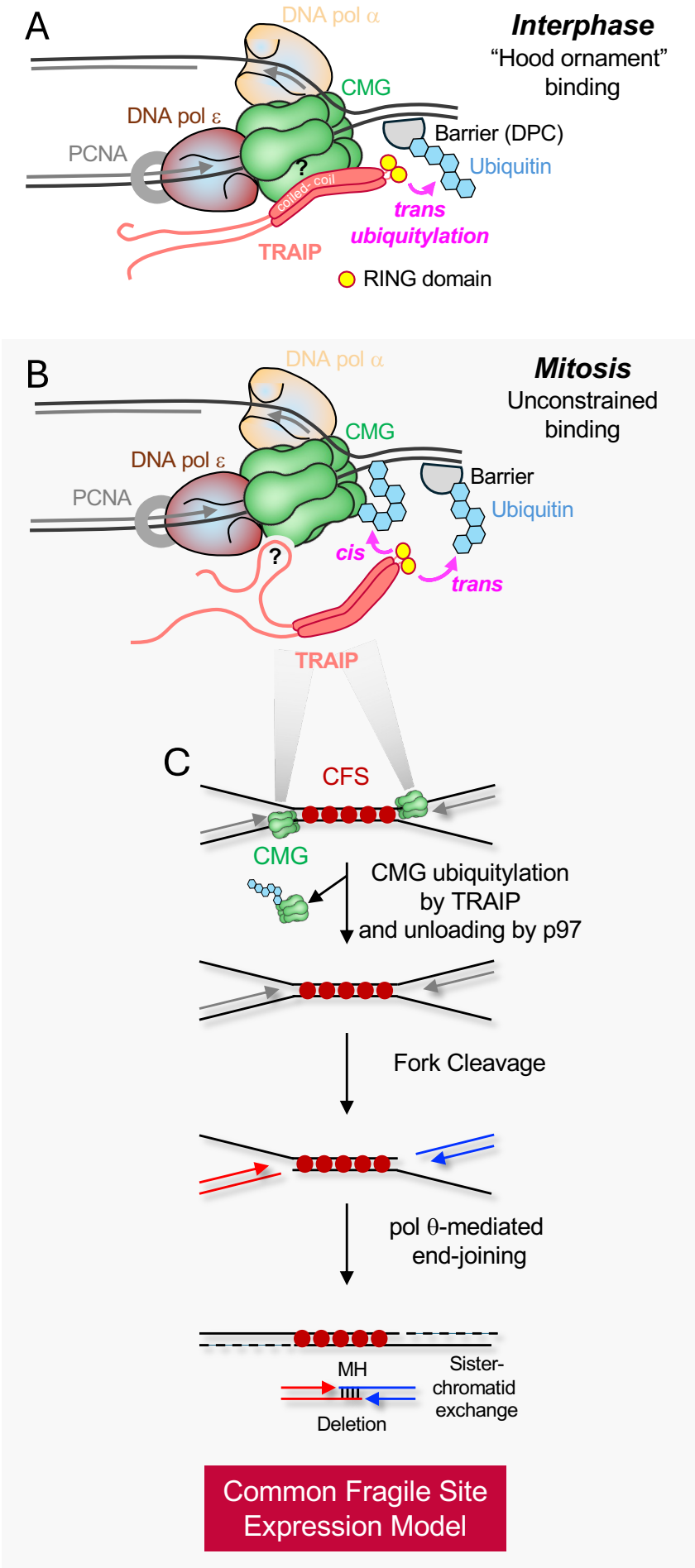

Figure S2

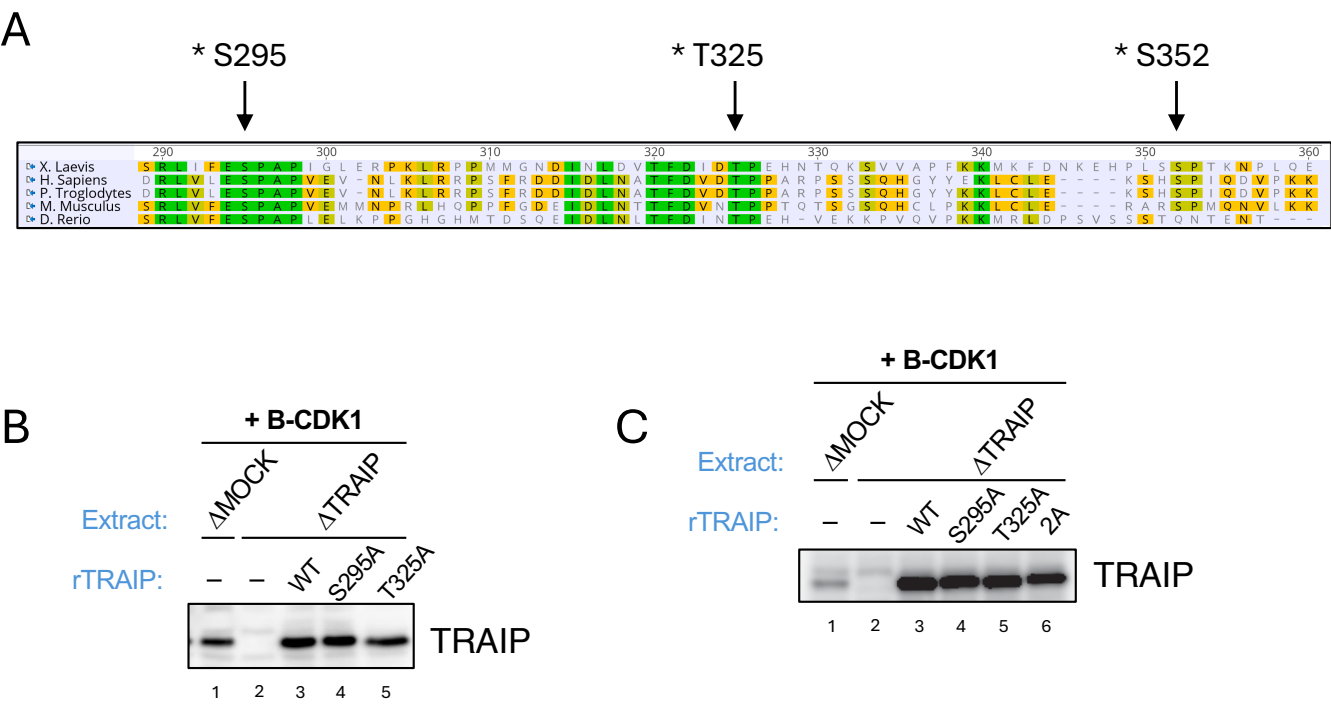

Figure S3

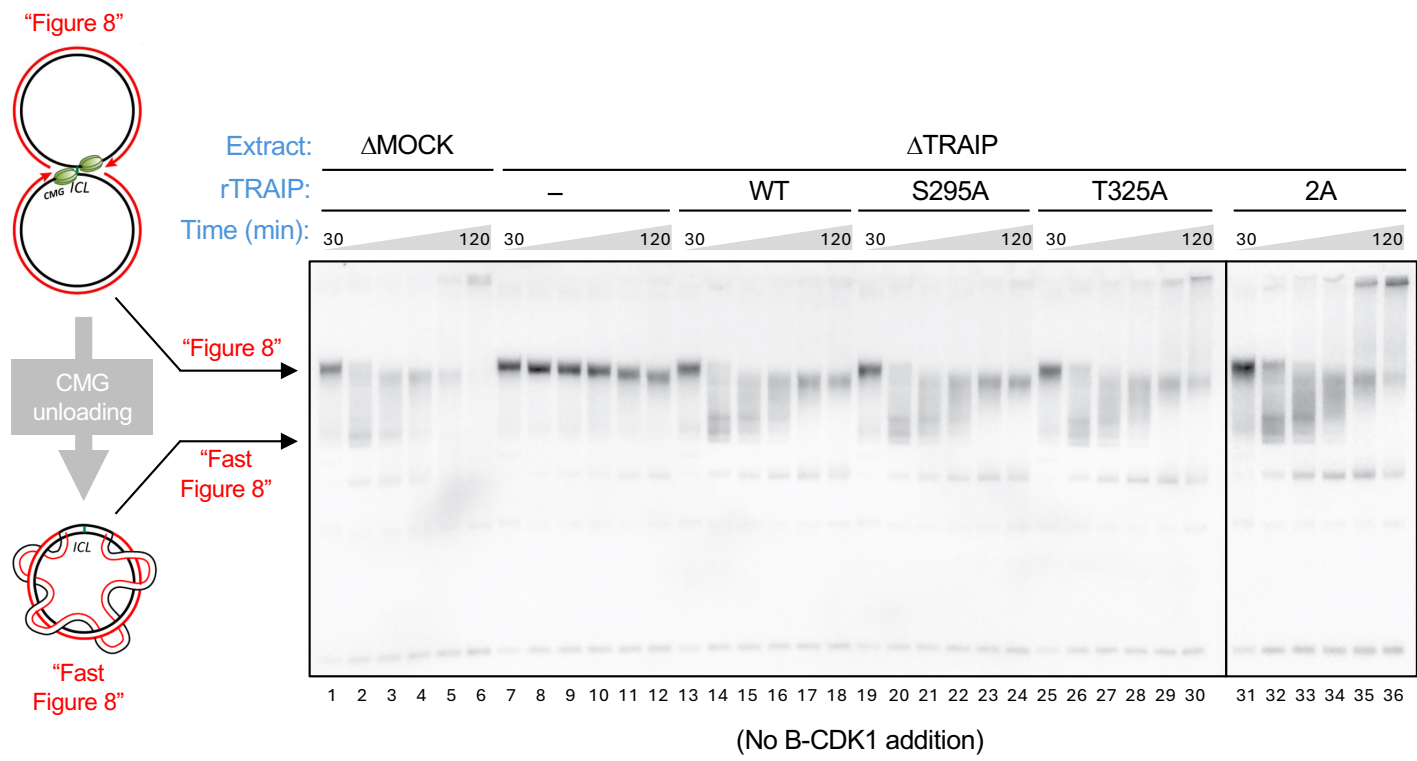

Figure S4

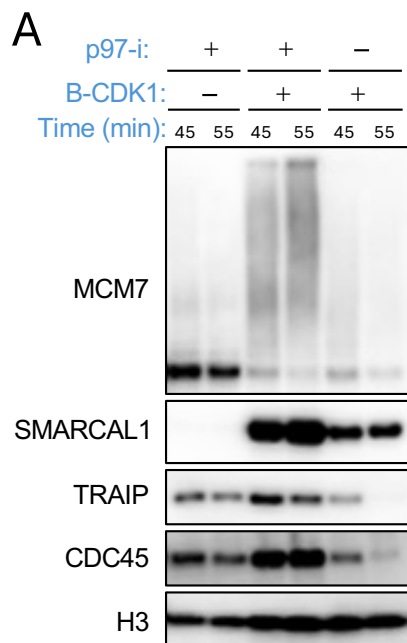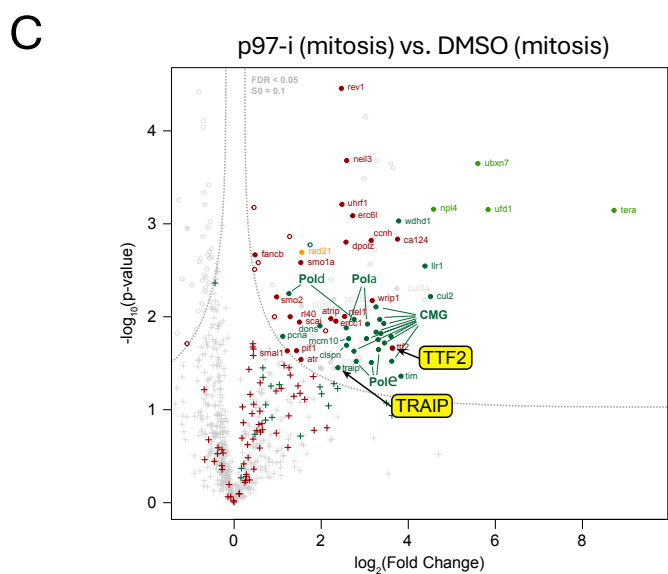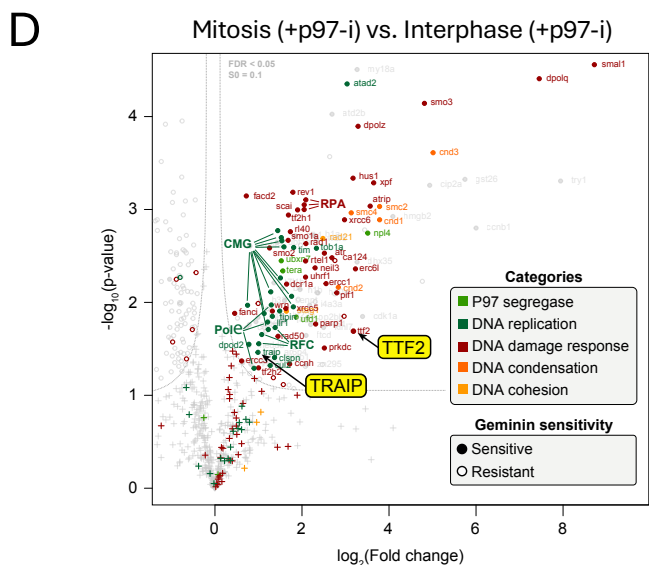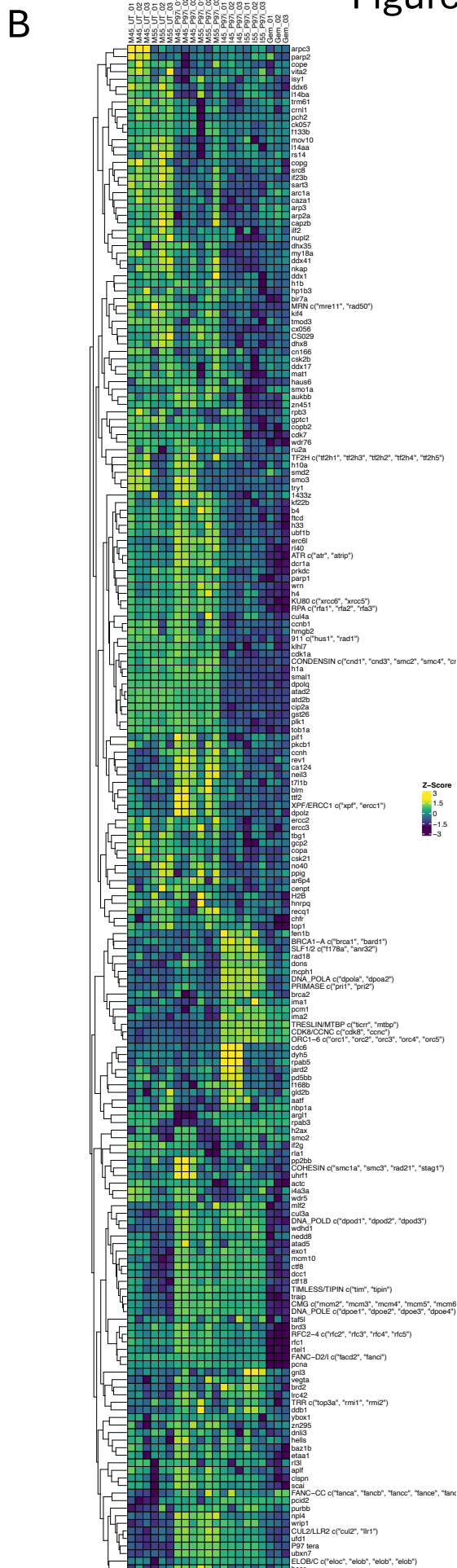

Figure S5

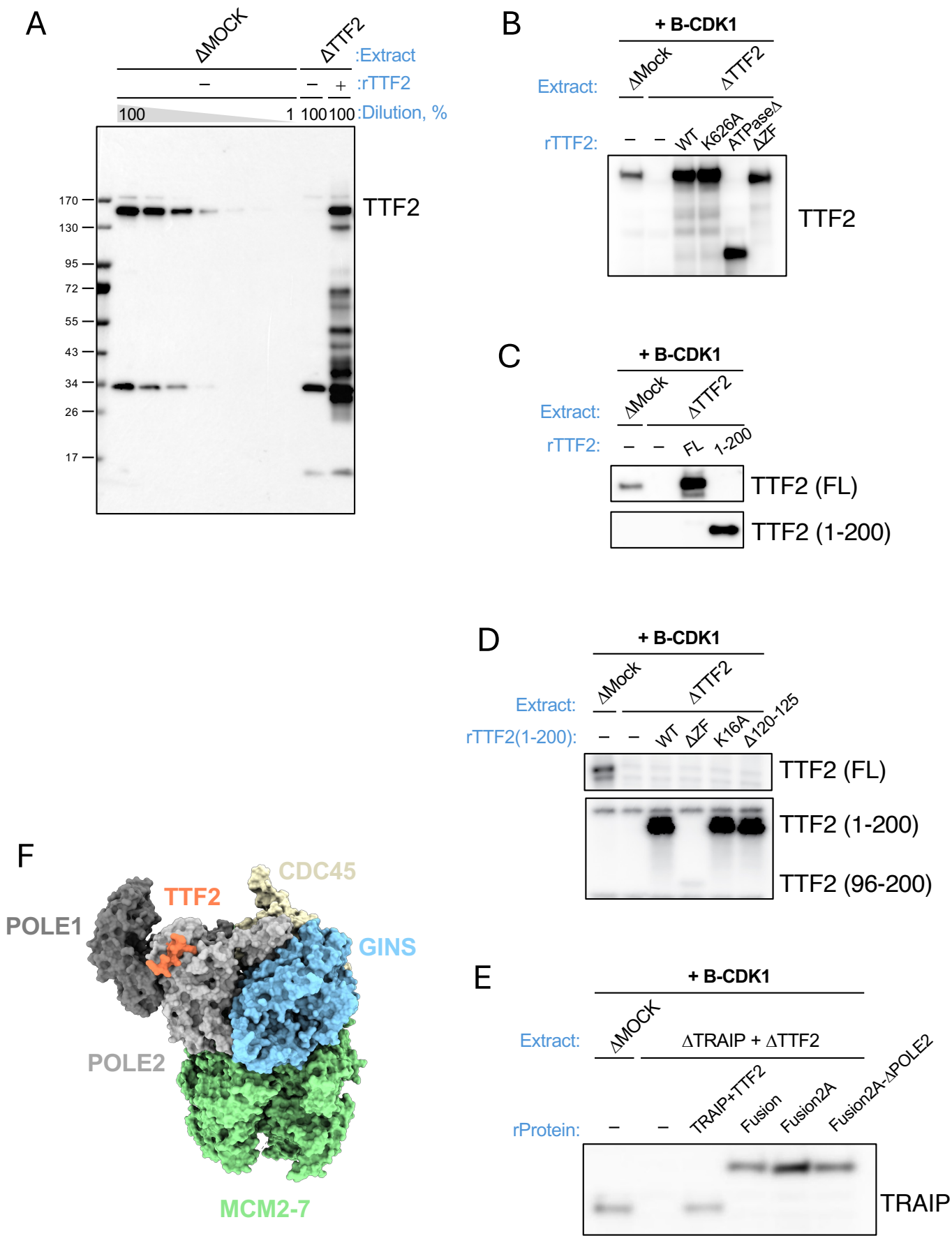

Figure S6

A

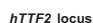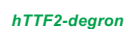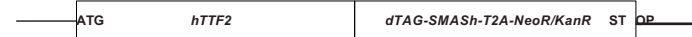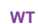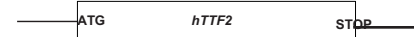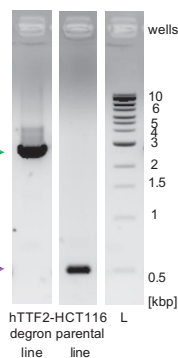

B

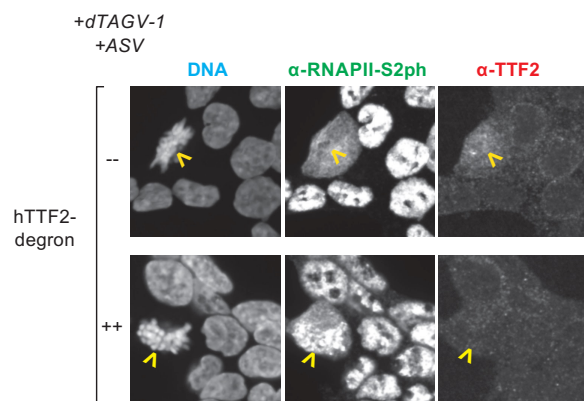

D

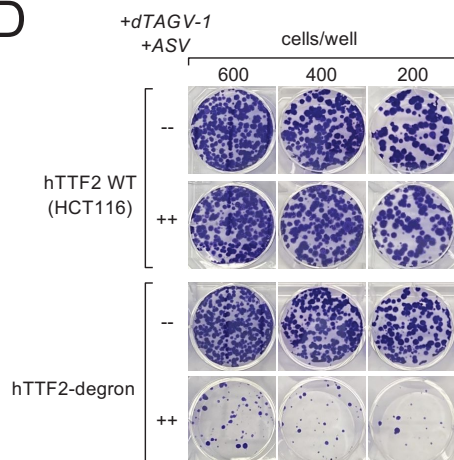

C

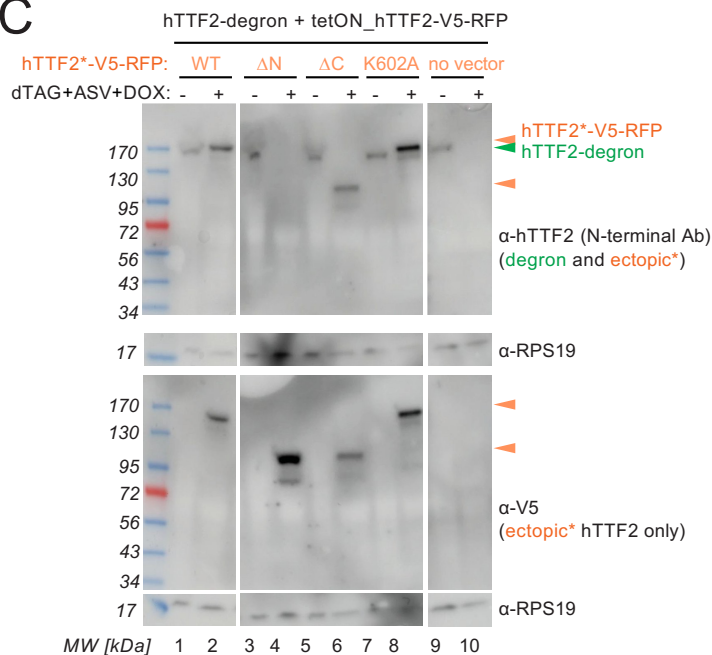

E

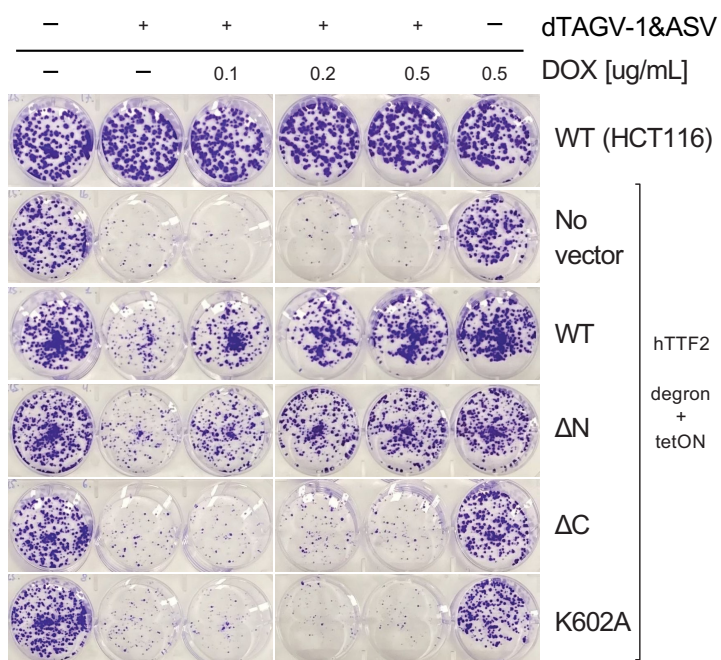

Supplement: 1 — Figure S1: Models for TRAIP replisome binding in the S and M phases (A) Interphase configuration of TRAIP on the replisome. The TRAIP dimer binds the replisome via an unknown mechanism (?) such that the catalytic RING domains (yellow circles) are constrained and can only ubiquitylate proteins ahead of the replisome in trans (“Hood ornament” model). In this configuration, TRAIP ubiquitylates proteinaceous barriers ahead of the fork such as covalent DNA protein cross-links (DPCs) or a CMG that resides on the other side of a DNA interstrand cross-link (not depicted), but it cannot ubiquitylate the CMG with which it travels. (B) Mitotic configuration. TRAIP is flexibly attached to the replisome via an unknown mechanism (?). In this state, the catalytic RING domain is not constrained and can also ubiquitylate the hosting CMG in “cis.” (C) Model for common fragile site (CFS) expression. If replisomes cannot copy a difficult to replicate locus (CFS) before cells enter mitosis, the stalled CMGs undergo TRAIP-dependent ubiquitylation and p97-dependent unloading, which deprotects the forks and induces symmetric fork cleavage. The broken chromosomes (red and blue) are ligated to each other by pol θ-mediated end-joining, which leads to a small deletion with microhomology (MH) at the breakpoint and sister chromatid exchange, as seen during common fragile site expression. Figure S2: Evidence for CDK phosphorylation of TRAIP. (A) Sequence alignment of vertebrate TRAIPs. The three conserved CDK sites are indicated. (B) Extracts used for the experiment in Fig. 1D were blotted for TRAIP. (C) Analogous to (B), but for Fig. 1E&F. Figure S3: TRAIP CDK sites are not required for interphase TRAIP function. (A) A plasmid containing a cisplatin DNA inter-strand crosslink was replicated in the indicated interphase egg extracts containing [α−32P]dATP. At 15, 30, 60, 90 and 120 minutes, samples were separated on a native agarose gel and visualized by autoradiography. After replication initiati [file NIHPP2024.11.30.626186V1-supplement-1.pdf]
